# Testing the effects of topical skin care products on Tumor Treating Fields (TTFields) adhesiveness of arrays and delivery of electric currents

**DOI:** 10.1007/s00520-025-10085-9

**Published:** 2025-11-01

**Authors:** Maya Sadovnik, Sewar Zbidat, Mariell Sellevoll, Stav Edelstein, Gitit Lavy-Shahaf, Roni Blatt, Itai Tzchori, Adi Haber, Moshe Giladi, Uri Weinberg, Yoram Palti

**Affiliations:** 1 Novocure Ltd, Haifa, Israel; 2 Novocure GmbH, Baar, Switzerland

**Keywords:** Tumor Treating Fields (TTFields) therapy, Skin adverse events, Skin care management, Topical skin care products

## Abstract

**Purpose:**

Tumor Treating Fields (TTFields) therapy is an approved cancer treatment, delivered to patients continuously via arrays applied to the skin surrounding the tumor region. The delivery of treatment by skin-adherent arrays creates an inherent risk of skin irritation. Implementing proactive measures for skin care with appropriate agents is necessary to maintain patient quality-of-life and increase device usage for better treatment effectiveness. The goal of this study was to examine the effects of commercially available skin care products on array adhesiveness and electric current delivery, to determine which may be used to alleviate device-related skin irritation without compromising treatment effectiveness.

**Methods:**

We tested skin care products that are customary for the treatment of skin adverse events (AEs), including skin barriers, topical corticosteroids, anti-itching products, wound healing agents, and topical antimicrobials. We also tested moisturizers, recommended for preserving good skin health. We performed a wide screen on 34 compounds in animal models, and then validated the results in humans for 8 of the compounds.

**Results:**

The preclinical tests identified 24 compounds that did not interfere with TTFields delivery, coming from the various medical use groups. Those that significantly reduced array adhesiveness and electrical conduction contained an electrical insulator/lipid component as the major ingredient. The results from the human study were in concordance with the preclinical results.

**Conclusions:**

This study identified, under controlled conditions, a selection of commercially available skin care products that do not compromise TTFields delivery and hence may serve as candidates for managing skin AEs in TTFields-treated patients.

**Supplementary Information:**

The online version contains supplementary material available at 10.1007/s00520-025-10085-9.

## Introduction

Tumor Treating Fields (TTFields) are electric fields that disrupt cellular processes critical for cancer cell viability and tumor progression. TTFields therapy is approved for treatment of patients with recurrent glioblastoma (GBM), newly-diagnosed GBM (concurrent with maintenance temozolomide), unresectable, locally advanced or metastatic pleural mesothelioma (concomitant with pemetrexed and a platinum-based agent), and metastatic non-small cell lung cancer (NSCLC; together with PD-1/PD-L1 inhibitors or docetaxel) following progression on or after platinum based chemotherapy [1–3]. Most recently, two pivotal trials demonstrated that TTFields therapy prolonged time to intracranial progression for patients with brain metastases from NSCLC relative to best supportive care (METIS trial, NCT02831959) [4] and extended overall survival for patients with unresectable, locally advanced pancreatic adenocarcinoma treated in the first-line setting with TTFields therapy concomitant with gemcitabine and nab-paclitaxel relative to treatment with chemotherapy alone (PANOVA-3 trial, NCT03377491) [5].

**Fig. 1:**
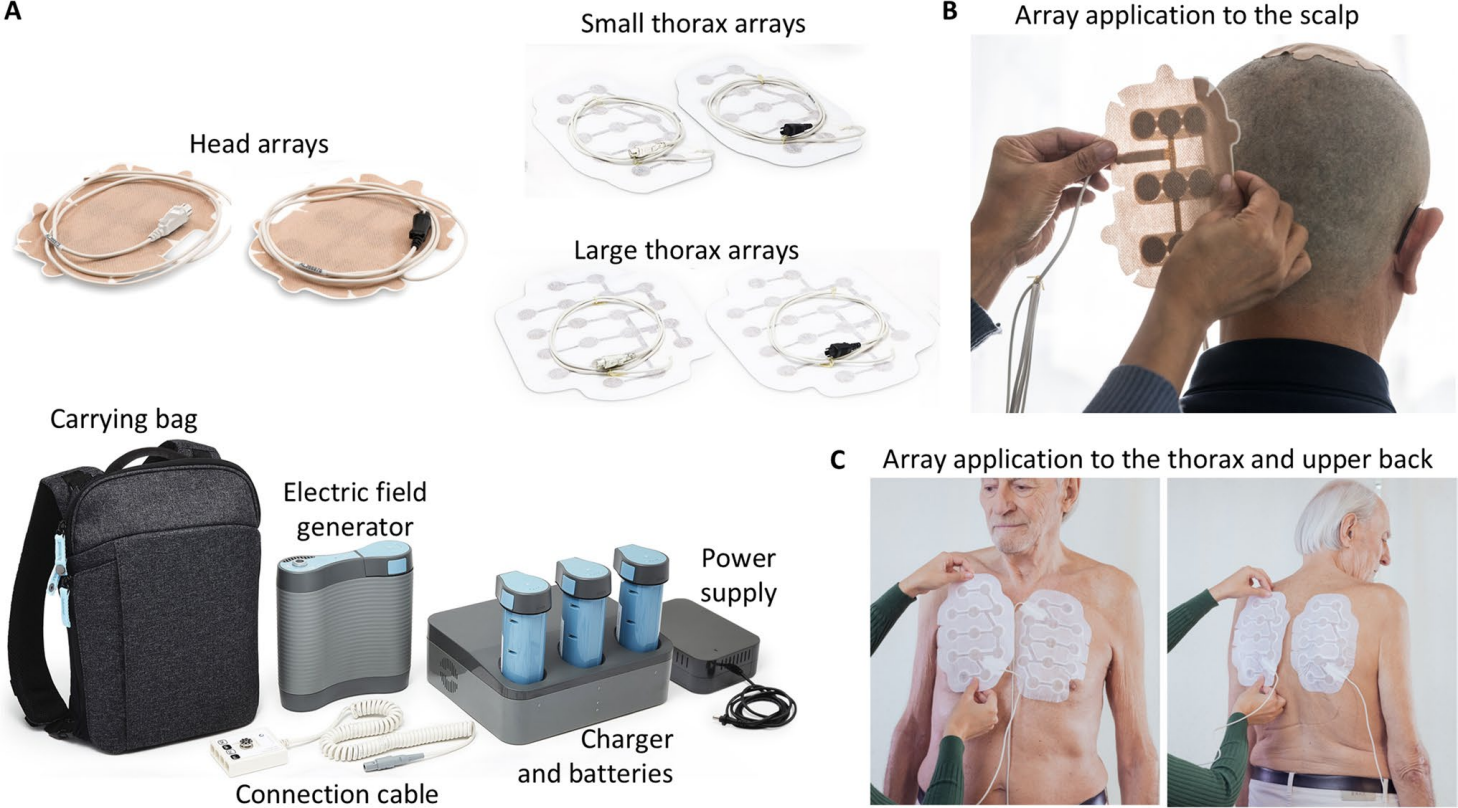
The TTFields device. The components of the device used for TTFields therapy delivery: head arrays, small/large thorax arrays, carrying bag, electric fields generator, connection cable, charger and batteries, power supply. TTFields array placement on the scalp (**B**) and on the thorax (**C**). The models depicted in the figure are actors and not patients

TTFields therapy is delivered to patients continuously using a portable electric field generator (Fig. 1). The treatment is applied non-invasively via two pairs of arrays, placed on the patient’s skin surrounding the tumor region: on the scalp for treatment of GBM and brain metastasis, on the thorax for treatment of mesothelioma and NSCLC, and on the lower torso in clinical trials for treating pancreatic, liver, gastric, and other cancer types residing in the abdomen [2, 6]. A meta-analysis by Ballo et al. of GBM studies reporting on device usage time demonstrated an association between prolonged overall survival and a usage rate of ≥ 75% (18 h/day) [7].

The delivery of prolonged continuous treatment by skin-adherent arrays creates an inherent risk of skin irritation due to poor ventilation and excessive sweating underneath the arrays, the repeated shaving and adhesive tape tearing from the skin upon array replacements, and due to pressure and friction from the device during daily body movement.

Indeed, the main TTFields device-related adverse events (AEs) reported in clinical trials and real-world evidence has been mild to moderate skin irritation under the arrays [3, 4, 8, 9]. Early skin AEs do not impose significant treatment disturbance. However, in rare cases, improper monitoring and management can lead to exacerbation, which negatively impacts patient‘s quality-of-life (QoL) and compromises patient adherence to treatment. Severe forms of skin irritation may require treatment interruptions until resolution or even treatment discontinuation, reducing device usage time. The skin AEs may also affect practical aspects of treatment delivery, such as adhesion of the arrays to the skin.

Prophylactic strategies for preventing TTFields device-related skin AEs include maintaining skin health using moisturizers to ensure hydration and skin barriers to prevent damage, leaving the skin uncovered, and repositioning the arrays upon replacements. Management strategies for preventing exacerbation include regular monitoring with dermatologic care as needed, including skin barriers, topical corticosteroids, anti-itching products, wound healing agents, antiseptic solutions, or anti-microbials [10–12].

Since the electric fields of TTFields therapy are delivered into the body through the array–skin interface, special attention should be given to compounds applied topically at the location of the arrays to avoid compromising treatment effectiveness. Of note, TTFields application may cause heating under the arrays, hence the device is designed to measure the temperature and respond to prevent overheating. Device responses may include adjustments to reduce applied currents or even the complete cessation of current delivery (i.e. treatment break). Since treatment effectiveness is dependent on field intensity (i.e. electric current) and device usage (i.e. daily time on treatment), topical skin care products that introduce high resistance to the array–skin interface may cause excessive temperature elevation, potentially interfering with treatment effectiveness [7]. Overall, skin AEs should be managed to preserve patient QoL and allow continued treatment; however, attention should be given to avoid topical skin care products that may compromise treatment effectiveness.

The goal of this study was to examine the effects of commercially available topical skin care products on the adhesiveness of TTFields arrays and delivery of TTFields electric current, and to determine which may be considered by the treating physician to alleviate device-related skin irritation without compromising treatment effectiveness. An animal-based model was used for initial screening of a large group of compounds, and subsequent human studies validated the findings for a few selected products.

## Methods

### Topical products

Thirty-four commercially available topical skin care products intended for various medical uses were examined in this study, including topical antimicrobials, topical corticosteroids, anti-eczema/anti-itching products, skin barriers/wound healing agents, and moisturizers. Compounds with different application methods were used, mainly creams, gels, sprays, and ointments. Table S1 lists the specific products, including details of their medical use, application method, and ingredients.

### Animal care

Rats were kept at 20–24 °C and 40–70% humidity, with temperature and humidity recorded twice a day. A 12-h light–dark cycle and 12 fresh air exchanges per hour were maintained. Rats received a standard diet with food and water ad libitum. All animal studies were approved by the Animal Experimentation Committee according to the Ministry of Health by the Israel National Council for Animal Experimentation (approval number: NPC-No-IL-2302–199-4) and performed in accordance with guidelines and regulations for the care of laboratory animals.

### Mechanical functionality testing of topical skin care products in rats

Male, 7–8-week-old Sprague Dawley rats (SD, body weight over 200 gr) (Figure S1A for study scheme) were anesthetized with isoflurane, and the fur from their back removed using a trimmer and depilating cream (Veet). An anesthetized rat was then placed on its abdomen on the platform of a LTCM-100 motorized force tester (Chatillon, Figure S1B). A human head array was pre-cut into 3 equal parts, and one part was applied to the rat back and left untouched for 2.5 min to allow full adherence to the skin (Figure S1C). A strip of adhesive was used to connect the dorsal edge of the array to the machine’s motorized arm (Figure S1D). Peeling was performed by the arm pulling the adhesive strip with a speed rate of 1.5 cm/min (Figure S1E), during which a digital force gauge (Chatillon) measured the generated force. The array was removed, and the topical product was applied to the rat’s back according to the manufacturer’s instruction and left untouched for 5 min to allow full absorption into the skin. A new part of the human head array was then applied on the rat back at the same position, and connected to the machine arm with an adhesive strip. Peeling force was measured as described above. Each topical product was tested on one rat. The percentage change in force required for peeling with each topical product relative to the force without the product was calculated.

### Electrical functionality testing of topical skin care products in rats

Male, 7–8-week-old SD rats (body weight over 200 gr) (Figure S2A for study scheme) were anesthetized with isoflurane, and the fur from their torsos removed using a trimmer and depilating cream (Veet). Rat torso TTFields arrays (specifically designed for rats, but identical in composition to the arrays used to deliver TTFields to patients) were assembled on the depilated rat torso (Figure S2B-C). The 2 dorsal arrays were located 1 cm from each side of the spinal cord, and the 2 ventral arrays at 5 mm from each side of the midline. The arrays were secured using a hypo-allergenic, medical-grade adhesive tape (Figure S2D-E). The arrays were then connected to the inovivo system (a system for delivering TTFields to animals [13, 14]), and TTFields (200 kHz) were applied continuously for 60 min (after reaching electric current steady state).

Next, the arrays were removed, and the topical product was applied to the rat’s torso according to the manufacturer’s instruction, and left untouched for 5 min to allow full absorption into the skin. New rat torso TTFields arrays were applied at the same position as described above. The arrays were then connected to the inovivo system, and TTFields (200 kHz) were applied continuously for 60 min (after reaching electric current steady state). For each skin care product, the experiment was performed also in the opposite order, first delivering TTFields with the topical product applied, and then delivering TTFields without the topical product. Each topical product was tested on three to four rats. The percentage change in current generated with the topical product relative to the current generated without the product was calculated for each rat. Mean and standard deviation were calculated, and statistical significance examined with a paired Student T-test.

### Electrical functionality testing of topical skin care products in humans

**Fig. 2:**
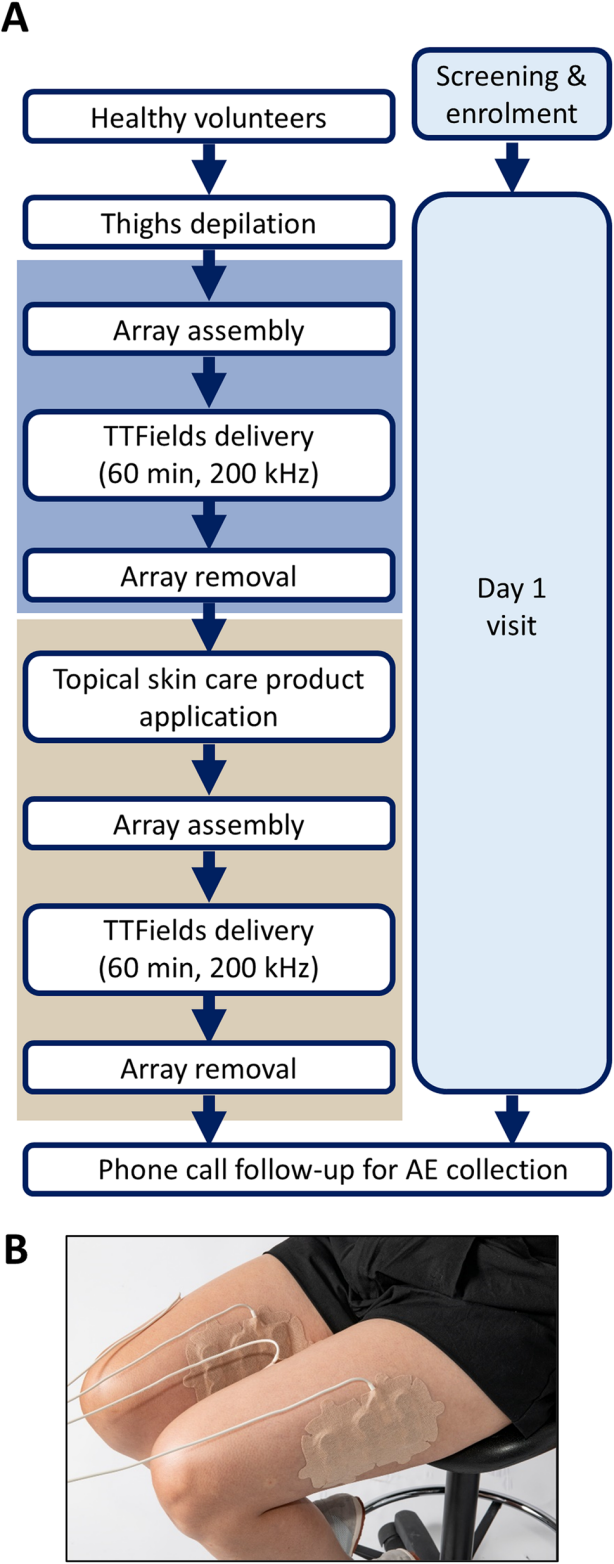
The clinical study. Clinical study scheme (**A**) and array placement on the thighs during the study (**B**)

A single arm, open label, single center pilot study in healthy volunteers was conducted between December 2023 and August 2024 at the Rambam Medical Center, Haifa, Israel (Fig. 2A for study scheme). The ethics committee reviewed and approved the protocol. Inclusion criteria were: aged ≥ 18 years; and signed written informed consent. Exclusion criteria were: presence of implantable electrical medical devices; known allergies to medical adhesives or hydrogel; skin lesions where the arrays were planned to be placed or chronic skin disease; known acute or significant chronic disease as determined by the investigator; and pregnant or breastfeeding. Twenty-six healthy volunteers who provided informed consent for the study participated in 1 day of tests and 30 days of safety follow-up.

On the test day, the volunteer’s thighs were shaved, and one pair of human head arrays was applied at the middle of each thigh (with the lower part of the array 9–13 cm above the knee, Fig. 2B). The arrays were then connected to the TTFields-delivering device, and TTFields (200 kHz) were applied continuously for 60 min (after reaching electric current steady state). Next, the arrays were removed, and one topical product was applied according to the manufacturer’s instruction to each of the volunteer’s thighs and left untouched for 10–15 min to allow full absorption into the skin. New head arrays were applied on the volunteer’s thighs at the same position as before. The arrays were then connected to the TTFields-delivering device, and TTFields (200 kHz) were applied continuously for 60 min (after reaching electric current steady state). A total of eight topical skin care products were tested, seven of them tested on seven to eight volunteers. The remaining one compound served as negative control and was tested on only one volunteer. The percentage of change in current generated with the topical product versus without it was calculated for each volunteer. Median and interquartile range (IQR) were calculated, and significance examined with a paired Wilcoxon signed rank test.

## Results

### Feasibility testing of topical skin care products in rats

We examined thirty-four common topical skin care products for their feasibility of use together with TTFields treatment (Table S1 for list of ingredients). Since good skin adherence is required for efficient treatment delivery, we first tested the effect of the topical skin care products on array attachment strength, by measuring the change in the force needed to peel off the arrays from the shaved rat back when applying the skin care product relative to that needed when no topical product was applied (Table 1). Ten of the tested compounds substantially reduced the required force by 40% or more (Baby Pasta, Bepanthen Plus, Calendula, Cicaplast, Fucidin, Gillette, Maalox, NewGel + E, Silverol, U Lactin). These products were therefore considered to interfere with array adherence to the skin, and were not tested for electrical functionality. For the twenty-four compounds that passed the peeling test, we next tested their effect on current delivery via the arrays, by measuring the change in current when applying the skin care product relative to that when no topical product was applied (Table 1). Four products significantly reduced the current delivered via the arrays (Cicalfate, Desitin, Kelo Cote, Staquis). Interestingly, these four compounds reduced the peeling force by 20–40%. Overall, twenty of the tested compounds were identified as passing the feasibility tests.

**Table 1:** Force and current change for the topical skin care products tested on rats

Topical Product	Medical Use	Application Method	Force Change (%)	Current		Pass/Fail
				Change (%)	p-value	
				[mean ± SD]		
Atoderm	Moisturizers	Cream	−5	−5.94 ± 4.79	0.082	Pass
Baby Pasta	Skin Barriers/Wound Healing	Ointment	−63.2	NA	NA	Fail
Bepanthen Plus	Topical antimicrobials	Cream	−79.9	NA	NA	Fail
Betacortene	Topical corticosteroids	Cream	38.9	1.79 ± 12.17	0.841	Pass
Calamine	Anti-Eczema/Anti-Itching	Suspension	109.9	−4.43 ± 3.92	0.106	Pass
Calendula	Skin Barriers/Wound Healing	Cream	−100	NA	NA	Fail
Cavilon	Skin Barriers/Wound Healing	Spray	341	1.29 ± 8.52	0.801	Pass
CeraVe	Moisturizers	Cream	43.2	−0.04 ± 14.77	0.882	Pass
Cicalfate	Skin Barriers/Wound Healing	Cream	−30.7	−22.72 ± 7.33	0.009	Fail
Cicaplast	Skin Barriers/Wound Healing	Cream	−100	NA	NA	Fail
Dermalibour +	Moisturizers	Foaming Gel	48.2	8.13 ± 11.86	0.266	Pass
Dermovate	Topical corticosteroids	Cream	226.7	−1.88 ± 11.78	0.743	Pass
Desitin	Skin Barriers/Wound Healing	Ointment	−38.9	−48.90 ± 4.85	0.0005	Fail
Elidel	Anti-Eczema/Anti-Itching	Cream	83.3	2.24 ± 8.10	0.719	Pass
Esenta spray	Skin Barriers/Wound Healing	Spray	78.6	−2.92 ± 10.63	0.568	Pass
Esenta wipes	Skin Barriers/Wound Healing	Wipes	204.7	−0.50 ± 3.48	0.701	Pass
Fucidin	Topical antimicrobials	Cream	−61.5	NA	NA	Fail
Gentatrim	Topical antimicrobials	Cream	4.9	4.30 ± 4.71	0.1608	Pass
Gillette (Sport truimph)	Antiperspirant	Gel	−77.1	NA	NA	Fail
Ialuset	Moisturizers	Cream	77.1	−0.64 ± 8.37	0.85	Pass
Kelo Cote	Skin Barriers/Wound Healing	Spray	−31.8	−27.31 ± 12.31	0.011	Fail
Lipikar	Moisturizers	Cream	4	0.98 ± 4.09	0.695	Pass
Locapred	Topical corticosteroids	Cream	1.9	0.79 ± 5.20	0.777	Pass
Maalox	Skin Barriers/Wound Healing	Cream	−49.1	NA	NA	Fail
Mupirocin	Topical antimicrobials	Ointment	−13.5	3.72 ± 5.17	0.242	Pass
Neriderm	Moisturizers	Cream	−32.7	−17.99 ± 5.80	0.013	Pass
NewGel + E	Moisturizers	Gel	−66.3	NA	NA	Fail
Secura	Skin Barriers/Wound Healing	Spray	176.4	−4.65 ± 5.92	0.21	Pass
SensiCare	Skin Barriers/Wound Healing	Spray	186.8	1.26 ± 12.29	0.986	Pass
Silverol	Topical antimicrobials	Cream	−61.6	NA	NA	Fail
Staquis	Anti-Eczema/Anti-Itching	Ointment	−22.6	−42.13 ± 3.91	0.0004	Fail
U Lactin	Moisturizers	Cream	−41.9	NA	NA	Fail
Xeracalm	Anti-Eczema/Anti-Itching	Cream	10.6	−0.63 ± 5.76	0.816	Pass
Zindaclin	Topical antimicrobials	Gel	122.9	4.32 ± 9.23	0.482	Pass

Force change – Change in force required for array peeling with and without topical product applied to the skin

Current change – Change in current generated by application of 200 kHz TTFields with and without topical product applied to the skin

NA – non applicable, as for compound that reduced the peeling force by 40% or more, change in electrical current was not tested

Compounds that did not reduce the peeling force by more than 40% and did not significantly reduce the electrical current were labelled Pass. Compounds that did not comply with these conditions were labelled Fail. Importantly, while it is advised to avoid the use of the compounds labelled Fail during the course of TTFields delivery, such compounds may still be applied during treatment breaks as long as they are removed before array placement

In our test panel, topical skin care products that significantly reduced the force or current were those that contain, as the major ingredient, compounds that are electric insulators (zinc oxide, aluminum-magnesium oxide, polymerized siloxane) or are hydrophobic (mineral/seed oil, glycerol, paraffin, Vaseline, or petrolatum). Water and silicon-based topical skin care products did not significantly reduce the force or current. Of note, compounds from all medical use groups could be found within the skin care products that did not reduce the force nor the current. Of the four tested ointments, the only one that did not reduce force or current contained a hydrophilic-based vehicle, polyethylene glycol, rather than hydrophobic substances.

### Feasibility testing of topical skin care products in humans

We selected eight of the twenty-four compounds that passed the peeling test for examination in humans, seven that did not significantly reduce the current in the rat tests (Calamine, SensiCare, Dermovate, Secura, CeraVe, Esenta wipes, and Xeracalm), and one to serve as a negative control (Staquis). Median age of the study cohort was 36.5 years (range: 20–59), 46% of the participants were males, and median BMI was 24.8 kg/m^2^ (range: 17–44) (Table 2). Each investigational skin care product was tested on seven to eight participants (Table 3).

**Table 2:** Subjects’ Baseline characteristics

Covariate/Sex		Mean	Std	Min	Median	Max	N
Age (years)	All	36.3	10.3	20.0	36.5	59.0	26
	Male	36.0	11.0	23.0	33.5	59.0	12
	Female	36.5	10.0	20.0	38.5	51.0	14
BMI (kg/m^2^)	All	25.9	5.8	17.0	24.8	44.0	26
	Male	26.4	3.7	21.6	25.5	33.4	12
	Female	25.5	7.3	17.0	23.1	44.0	14

*Std* standard deviation, *Min* minimal value, *Max* maximal value, *N* number of participants

**Table 3:** Investigational topical skin care product disposition

Topical Product	Gender				All	
	Male		Female			
	N	%	N	%	N	%
Calamine	0	0	7	100.0	7	100.0
CeraVe	4	50.0	4	50.0	8	100.0
Dermovate	0	0	7	100.0	7	100.0
Esenta Wipes	6	85.7	1	14.3	7	100.0
Secura	5	71.4	2	28.6	7	100.0
SensiCare	4	57.1	3	42.9	7	100.0
Xeracalm	3	42.9	4	57.1	7	100.0
Total tests^*^	22	44.0	28	56.0	50	100.0

*N* number of tests. *The number of total tests is double the number of participants since two products were tested per each participant, one on each thigh

**Fig. 3:**
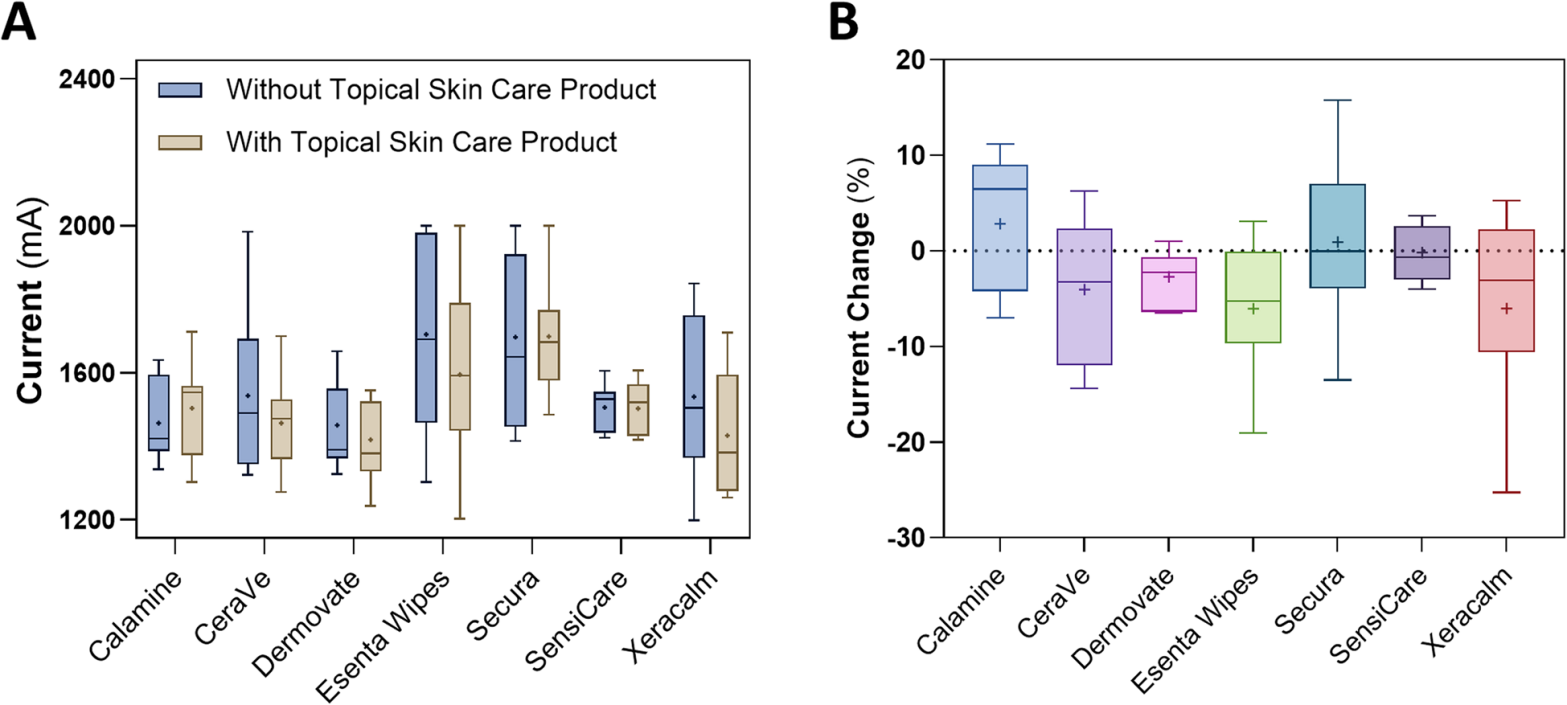
Current and current change for the topical skin care products tested on humans. TTFields (200 kHz) currents without (blue) and with (grey) application of topical skin care products to the thigh of clinical study participants (**A**), and the percent change of current generated with and without topical product applied to the skin (**B**). The boxes represent the interquartile range, the whiskers extend between the group minimum and maximum values, the horizontal line indicates the group median, and the plus sign denotes the group mean

The current measured across participants, with and without the various topical products, was between 1200 and 2000 mA (Fig. 3). For Calamine (median: 6.5%; IQR: −4.2% to 9.0%; p-value: 0.267), the majority of participants experienced an increase in current. With SensiCare (median: −0.7%; IQR: −3.0% to 2.6%; p-value: 0.937), while approximately 50% of the participants experienced a current decrease, variability was low and peak current reduction was less than 4%. Dermovate (median: −2.3%; IQR: −6.4% to −0.6%; p-value: 0.078) caused a decrease of current in almost all participants, though with low variability and a peak current reduction of less than 7%. Secura (median: 0%; IQR: −3.9% to 7.0%; p-value: 1) exhibited a current decrease in about 50% of participants, that exceeded 10% for 25% of participants. For CeraVe (median: −3.2%; IQR: −11.9% to 2.3%; p-value: 0.195), Esenta wipes (median: −5.2%; IQR: −9.7% to 0%; p-value: 0.047), and Xeracalm (median: −3.1%; IQR: −10.6% to 2.2%; p-value: 0.156), approximately 75% of participants experienced a current decrease, that exceeded 10% for 25% of participants.

## Discussion

We tested commercially available topical skin care products, suggested for treating TTFields device-related skin AEs, for their effect on arrays adhesiveness and the delivery of the electric current. While several publications have presented guidance for management of device-related AEs based on the authors’ clinical experience [15, 16], the current study aimed to identify skin care products that can be used without compromising treatment effectiveness. We developed two high-throughput screening methods of topical skin care products in rats and then validated the results in humans for a few selected compounds.

We tested topical skin care products that had different medical uses and that are frequently recommended by dermatologists as prophylactic measures or for the treatment of device-related skin irritation. These products included topical antimicrobials, topical corticosteroids, anti-eczema/anti-itching products, skin barriers/wound healing agents, and moisturizers. Of note, we categorized the different examined compounds into five major groups based on the primary medical use of each. However, some of the compounds have more than one use and hence may also belong to another medical use group. Most of the compounds we tested were creams, as these are most convenient for patient use; however, we also tested a few compounds with other application methods, mainly gels, sprays, and ointments.

The preclinical tests identified products that did not interfere with TTFields treatment in each of the medical use groups. Topical skin care products that significantly reduced array adhesiveness and electrical currents contained an electrical insulator/lipid component (e.g. zinc oxide, aluminum-magnesium oxide, polymerized siloxane, mineral/seed oil, glycerol, paraffin, Vaseline, or petrolatum) as the major ingredient. Accordingly, ointments were most associated with a reduction in peeling force and electrical currents, as these are mainly based on hydrophobic vehicle components, whereas creams are an emulsion of oil and water, and gels and sprays are mainly water-based. However, this does not mean that such ingredients should be avoided altogether. Skin products may not interfere with TTFields delivery when these ingredients are not the dominant component.

For the eight topical skin care products tested clinically, good concordance with the preclinical data was observed: (1) The Staquis ointment that showed a major decrease in array peeling force and a significant reduction in the electrical current in the preclinical tests prevented skin adherence of the array in the human study; (2) The other topical products, which did not compromise array adherence nor significantly reduce the currents in the animal studies, demonstrated no significant decrease in currents in the human studies (except for Esenta wipes, which nonetheless was associated with current reduction of less than 10%). Specifically, Calamine was associated with an increase in current in the majority of participants. For SensiCare and Dermovate there was a mild decrease in current with a narrow range of responses, indicating consistent results across all patients, making their responses easier to predict. For most participants the decrease in the current was less than 10%, and the mean and median decrease in current were less than 5%.

Importantly, identification of topical skin care products that do not interfere with TTFields delivery across medical use groups provides flexibility in prescribing a wide selection of treatments tailored to the management of TTFields device-related skin AEs. Importantly, while it is advised to avoid the use of specific compounds that may reduce treatment effectiveness during the course of TTFields delivery, such compounds may still be applied during treatment breaks as long as they are removed before array placement. Previously, the benefit of a fractionated schema protocol introducing intentional treatment breaks to allow active preventing approaches with topical skin care products was described [17]. However, care should be given to the amount of time off treatment since survival benefit from TTFields increases with duration-of-use [7, 18].

The work described in this study has a few limitations. First, TTFields delivery was evaluated at a frequency of 200 kHz, while 150 kHz is also clinically relevant. As TTFields frequency does not affect array adhesiveness nor electric currents, the outcomes are not expected to change for a different TTFields treatment frequency. Additionally, incidence of skin AEs in clinical studies was independent of the applied frequency [3, 19–21], supporting the extrapolation of results at 200 kHz into other frequencies. Secondly, testing of the topical skin care products in humans was performed on the thighs for practical reasons, whereas TTFields are applied to the scalp, thorax, or abdomen in the therapeutic setting. While array adhesiveness and currents may be different on different body parts, the percentage changes are likely generalizable. Thirdly, although a broad panel of 34 topical products was tested, some clinically relevant agents (such as hydrocortisone) were not included, and should be tested in future work. Finally, testing was performed in healthy volunteers under short-term TTFields exposure, which may not fully reflect patients with TTFields device-related skin reactions or the effects of repeated, long-term use. Of note, the study was designed to examine technical endpoints of array adhesion and current delivery, without assessing clinical outcomes such as skin tolerability, adverse event severity, patient adherence, or skin care product effectiveness. Future studies in patients receiving TTFields therapy are warranted to confirm whether the findings translate to improved skin management and tolerability in the clinical setting.

Overall, prophylaxis for skin care has the potential to prevent or reduce TTFields device-related skin AEs. When skin AEs do occur, they should be managed to prevent exacerbation to more severe forms of skin AEs, using topical skin care products carefully selected so they will not compromise TTFields delivery. Implementing proactive measures for skin care with an appropriate agent are necessary to maintain patient QoL and increase device usage for better treatment effectiveness. Our study examined the feasibility of using commercially available topical skin care products during TTFields delivery. The concordance between the clinical and preclinical results confirms the use of the high throughput preclinical approach to extrapolate the feasibility of using topical skin care products that have not yet been tested on humans. The next step will be to test these topical skin care products on patients receiving TTFields therapy, to offer insights into the prevention of skin AEs.

## Supplementary Information

Below is the link to the electronic supplementary material.

Supplementary file1 (PPTX 755 KB)

Supplementary file2 (DOCX 36 KB)

## Data Availability

All data generated or analyzed during this study are included in this published article and its supporting material. Further inquiries can be directed to the corresponding author.
