# Supplementary material for: Testing the effects of topical skin care products on Tumor Treating Fields (TTFields) adhesiveness of arrays and delivery of electric currents
Source: Support Care Cancer. 2025 Nov 1;33(11):1008. doi: 10.1007/s00520-025-10085-9 (PMC12579660; doi:10.1007/s00520-025-10085-9)
Supplement: Supplementary file 1 — Supplementary file1 (PPTX 755 KB) [file 520_2025_10085_MOESM1_ESM.pptx]

## Slide 1
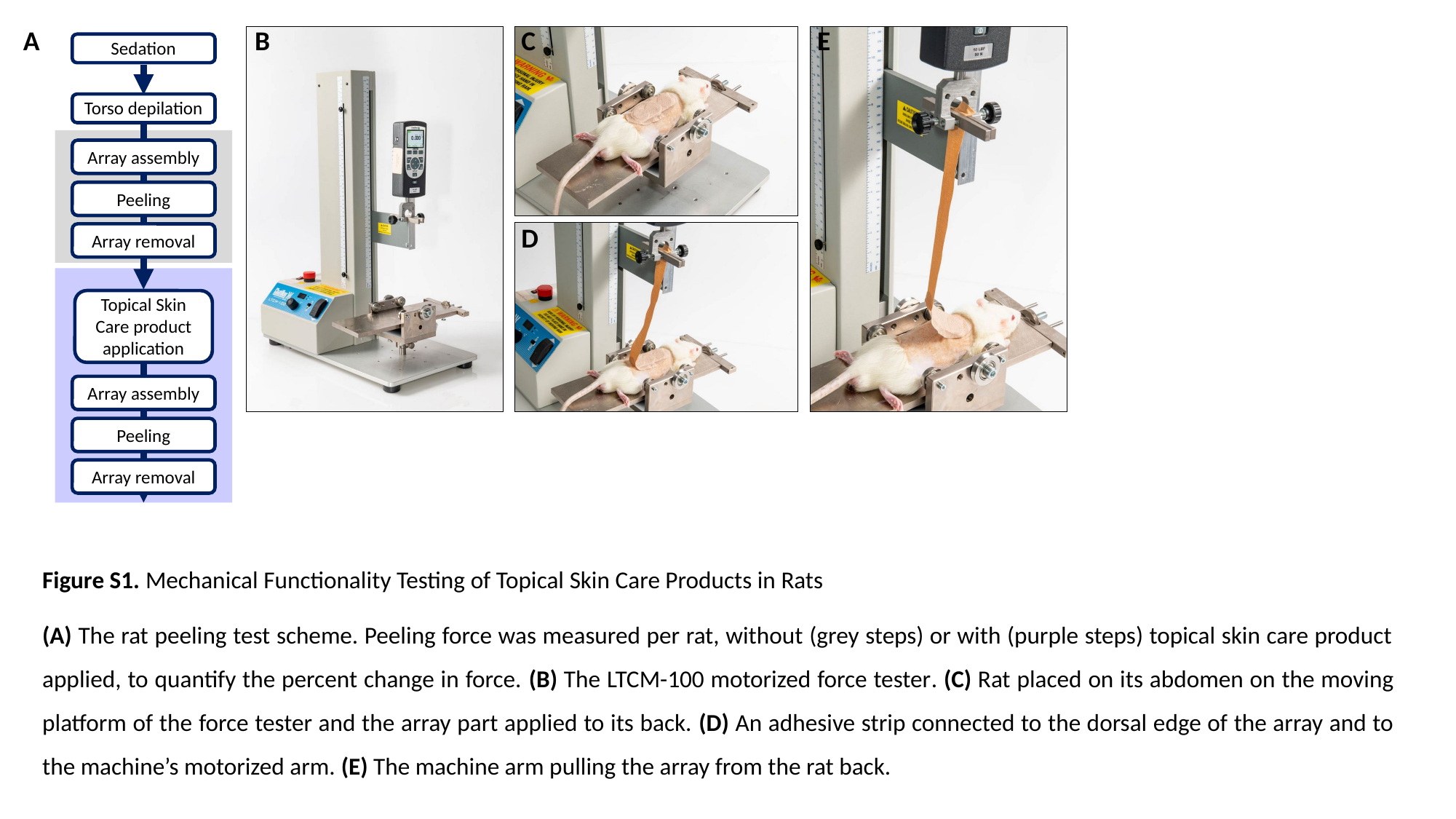

A
B
C
E
Sedation
Torso depilation
Array assembly
Peeling
Array removal
Topical Skin Care product application
Array assembly
Peeling
Array removal
D
Figure S1. Mechanical Functionality Testing of Topical Skin Care Products in Rats
(A) The rat peeling test scheme. Peeling force was measured per rat, without (grey steps) or with (purple steps) topical skin care product applied, to quantify the percent change in force. (B) The LTCM-100 motorized force tester. (C) Rat placed on its abdomen on the moving platform of the force tester and the array part applied to its back. (D) An adhesive strip connected to the dorsal edge of the array and to the machine’s motorized arm. (E) The machine arm pulling the array from the rat back.

## Slide 2
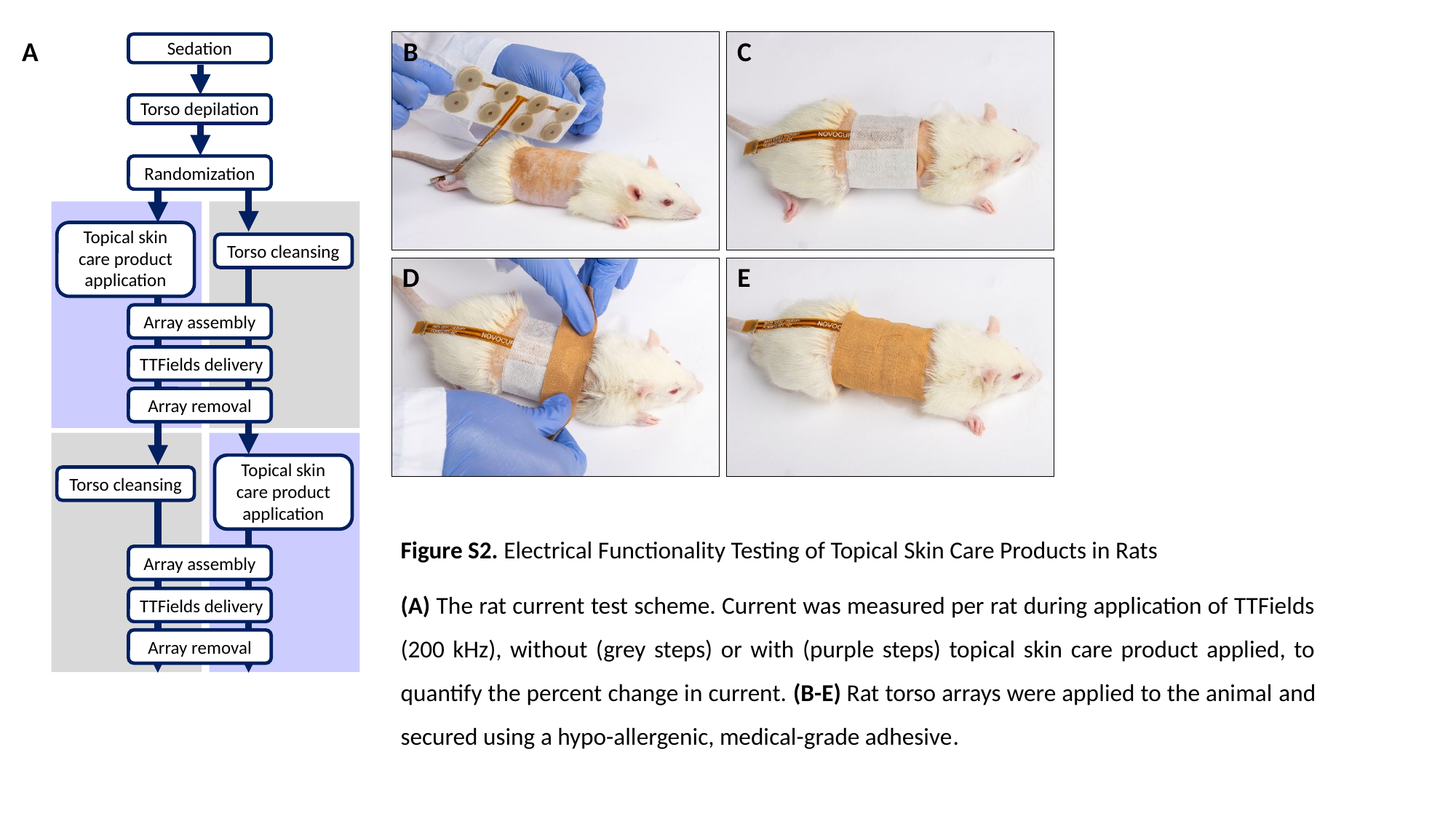

A
B
C
Sedation
Torso depilation
Randomization
Topical skin care product application
Torso cleansing
Array assembly
TTFields delivery
Array removal
Topical skin care product application
Torso cleansing
Array assembly
TTFields delivery
Array removal
D
E
Figure S2. Electrical Functionality Testing of Topical Skin Care Products in Rats
(A) The rat current test scheme. Current was measured per rat during application of TTFields (200 kHz), without (grey steps) or with (purple steps) topical skin care product applied, to quantify the percent change in current. (B-E) Rat torso arrays were applied to the animal and secured using a hypo-allergenic, medical-grade adhesive.
