# Supplementary material for: Testing the effects of topical skin care products on Tumor Treating Fields (TTFields) adhesiveness of arrays and delivery of electric currents
Source: Support Care Cancer. 2025 Nov 1;33(11):1008. doi: 10.1007/s00520-025-10085-9 (PMC12579660; doi:10.1007/s00520-025-10085-9)
Supplement: Supplementary file 2 — Supplementary file2 (DOCX 36 KB) [file 520_2025_10085_MOESM2_ESM.docx]

**Table S1. Ingredients of the Tested Topical Skin Care Products**

| **Topical Product** | **Medical Use** | **Application method** | **Ingredients** |
| --- | --- | --- | --- |
|  |  |  |  |
| Atoderm | Moisturizers | Cream | Water, Paraffinum Liquidum/​Mineral Oil/​Huile Minerale, Glycerin, Cetearyl Isononanoate, Glyceryl Stearate, PEG-100 Stearate, Myreth-3 Myristate, Steareth-21, Cyclopentasiloxane, Pentylene Glycol, Cyclohexasiloxane, Acrylates/​C10-30 Alkyl Acrylate Crosspolymer, Caprylyl Glycol, Cetyl Alcohol, Disodium EDTA, Palmitic Acid, Stearic Acid, Xylitol, Mannitol, Rhamnose, Sodium Hydroxide, Sodium Dehydroacetate, Xylitylglucoside, Anhydroxylitol, Niacinamide, Glucose, Fructooligosaccharides, Caprylic/​Capric Triglyceride, Laminaria Ochroleuca Extract |
| Baby Pasta | Skin Barriers/  Wound Healing | Ointment | Zinc Oxide 46.7% |
| Bepanthen Plus | Topical antimicrobials | Cream | Water, Lanolin, Paraffinum liquidum, Petrolatum, Panthenol, Prunus amygdalus dulcis oil, Cera alba, Cetyl alcohol, Stearyl alcohol, Ozokerite, Glyceryl oleate, Lanolin alcohol |
| Betacortene | Topical corticosteroids | Cream | Betamethasone valerate equivalent to 1 mg betamethasone (0.1 % w /w). Excipients/Inactive Ingredients: Methyl paraben 0.25 mg and Propyl paraben 0.15 mg as preservatives |
| Calamine | Anti-Eczema/  Anti-Itching | Suspension | Calamine 15%, Zinc Oxide 5%, Purified water, Glycerin, Bentonite, Sodium citrate, Liquefied phenol |
| Calendula | Skin Barriers/  Wound Healing | Cream | Water, Sesamum Indicum (Sesame) Seed Oil, Prunus Amygdalus Dulcis (Sweet Almond) Oil, Alcohol, Glyceryl Stearate SE, Lanolin, Beeswax (Cera Alba), Calendula Officinalis Flower Extract, Xanthan Gum, Fragrance (Parfum), Limonene, Linalool, Geranoil, Citral |
| Cavilon | Skin Barriers/  Wound Healing | Spray | Water, Diisooctyl Adipate, Acrylate Terpolymer, Cocos Nucifera (Coconut) Oil, PPG-15 Stearyl Ether, Glycerin, Isopropyl Palmitate, White Mineral Oil (Paraffinum Liquidum), Trimethylsiloxysilicate, Paraffin, Dimethicone, Phenoxyethanol, Benzoic Acid, Dehydroacetic Acid, Magnesium Sulfate |
| CeraVe | Moisturizers | Cream | Water, Glycerin, Cetearyl Alcohol, Caprylic/Capric Triglyceride, Cetyl Alcohol, Ceteareth-20, Petrolatum, Potassium Phosphate, Ceramide NP, Ceramide AP, Ceramide EOP, Carbomer, Dimethicone, Behentrimonium Methosulfate, Sodium Lauroyl Lactylate, Sodium Hyaluronate, Cholesterol, Phenoxyethanol, Disodium EDTA, Dipotassium Phosphate, Tocopherol, Phytosphingosine, Xanthan Gum, Ethylhexylglycerin |
| Cicalfate | Skin Barriers/  Wound Healing | Cream | Avène Thermal Spring Water, Caprylic/​Capric Triglyceride, Mineral Oil, Glycerin, Hydrogenated Vegetable Oil, Zinc Oxide, Propylene Glycol, Polyglyceryl-2 Sesquiisostearate, PEG-22/​Dodecyl Glycol Copolymer, Aluminum Sucrose Octasulfate, Aluminum Stearate, Beeswax, Copper Sulfate, Magnesium Stearate, Magnesium Sulfate, Microcrystalline Wax, Zinc Sulfate |
| Cicaplast | Skin Barriers/  Wound Healing | Cream | Water, Hydrogenated Polyisobutene, Dimethicone, Glycerin, Butyrospermum Parkii (Shea) Butter, Panthenol, Aluminum Starch Octenylsuccinate, Butylene Glycol, Propanediol, Cetyl Peg/Ppg-10/1 Dimethicone, Trihydroxystearin, Zinc Gluconate, Madecassoside, Manganese Gluconate, Silica, Aluminum Hydroxide, Magnesium Sulfate, Disodium Edta, Copper Gluconate, Citric Acid, Acetylated Glycol Stearate, Polyglyceryl-4 Isostearate, Tocopherol, Pentaerythrityl Tetra-Di-T-Butyl Hydroxyhydrocinnamate, Sodium Benzoate, Phenoxyethanol, Chlorhexidine Digluconate Titanium Dioxide |
| Dermalibour+ | Moisturizers | Foaming Gel | Water, Glycerin, Ceteareth-60 Myristyl Glycol, Decyl Glucoside, Zinc Coceth Sulfate, Disodium Laureth Sulfosuccinate, Avena Sativa (Oat) Leaf/​Stem Extract (Avena Sativa Leaf/​Stem Extract), Copper Sulfate, Zinc Sulfate, Citric Acid, Coco-Glucoside, Glyceryl Oleate, Hydrogenated Vegetable Glycerides Citrate, Laureth-3, Maleic Acid, Sodium Benzoate, Sodium Hydroxide, Tocopherol, Trisodium Ethylenediamine Disuccinate |
| Dermovate | Topical corticosteroids | Cream | Each 1 g contains 0.5 mg of clobetasol propionate (0.05% w/w). The other ingredients are cetostearyl alcohol, glyceryl monostearate 40-55, arlacel 165, beeswax substitute 6621, propylene glycol, chlorocresol, sodium citrate, citric acid and purified water |
| Desitin | Skin Barriers/  Wound Healing | Ointment | Zinc Oxide 13%, water, mineral oil, petrolatum, beeswax, dimethicone, sorbitan sesquioleate, microcrystalline wax, PEG-30 dipolyhydroxystearate, aloe barbadensis leaf extract, glycerin, tropolone, tocopheryl acetate, 1,2-hexanediol, caprylyl glycol, magnesium sulfate, potassium hydroxide, phenoxyethanol |
| Elidel | Anti-Eczema/  Anti-Itching | Cream | Each gram of ELIDEL Cream 1% contains 10 mg of pimecrolimus in a whitish cream base of benzyl alcohol, cetyl alcohol, citric acid, mono- and di-glycerides, oleyl alcohol, propylene glycol, sodium cetostearyl sulphate, sodium hydroxide, stearyl alcohol, triglycerides, and water. |
| Esenta spray | Skin Barriers/  Wound Healing | Spray | Silicone Blend |
| Esenta wipes | Skin Barriers/  Wound Healing | Wipes | Purified water, Polyvinylpyrrolidone, Glycerin. Propylene glycol. |
| Fucidin | Topical antimicrobials | Cream | The active ingridient is fusidic acid. 1 gram of Fucidin cream contains 20 mg of fusidic acid. The other ingredients are butylhydroxyanisole (E320), cetyl alcohol, glycerol, liquid paraffin, polysorbate 60, potassium sorbate, purified water, all-rac-α-tocopherol, hydrochloric acid and white soft paraffin. |
| Gentatrim | Topical antimicrobials | Cream | Each gram contains Gentamicin Sulfate USP equivalent to 1 mg gentamicin base in a cream base containing stearic acid, propylene glycol monostearate, isopropyl myristate, polysorbate 40, propylene glycol, sorbitol solution and purified water with methylparaben and butylparaben as preservatives |
| Gillette  (Sport truimph) | Antiperspirant | Gel | Aluminum Zirconium Octachlorohydrex Gly 16% (Anhydrous) - Antiperspirant. Inactive Ingredients: Water, Alcohol Denat., Cyclopentasiloxane, Propylene Glycol, Dimethicone, Trisiloxane, Calcium Chloride, PEG/PPG-18/18 Dimethicone, Fragrance. |
| Ialuset | Moisturizers | Cream | Hyaluronic Acid (Sodium Hyaluronate), Cetearyl Glucoside, Oleic Acid Decylester, Cetearyl Alcohol, Sodium Lauryl Sulfate, Sodium Cetearyl Sulfate, Glycerol, Liquid Sorbitol, ​Methylal, Propyl Parahydroxybenzoate, Trisodium Citrate Dihydrate, Water |
| Kelo Cote | Skin Barriers/  Wound Healing | Spray | Polysiloxanes, Silicon Dioxide |
| Lipikar | Moisturizers | Cream | Water, Butyrospermum Parkii Butter/Shea Butter, Glycerin, Dimethicone, Niacinamide, Paraffinum Liquidum/Mineral Oil, Cetearyl Alcohol, Brassica Campestris Oleifera Oil/Rapeseed Seed Oil, Ammonium Polyacryldimethyltauramide/Ammonium Polyacryloyldimethyl Taurate, Peg-100 Stearate, Glyceryl Stearate, Peg-20 Methyl |
| Locapred | Topical corticosteroids | Cream | Desonide |
| Maalox | Skin Barriers/  Wound Healing | Cream | Aqua cream and Aluminum-magnesium Hydroxide 1:1 |
| Mupirocin | Topical antimicrobials | Ointment | Mupirocin, Polyethyleme glycol |
| Neriderm | Moisturizers | Cream | Alcohol Stearyl, Stearate 40 Polyoxyl, Heavy Liquid Paraffin, White Vaselin 980, Carbopol, Dihydrate Disodium Edetate Parahydroxy Methyl, Hydroxide Sodium, Benzoate Parahydroxy Propyl, Benzoate |
| NewGel+ E | Moisturizers | Gel | Polymerized Siloxanes (Silicone Gel), Tocopherol (Vitamin E) |
| Secura | Skin Barriers/  Wound Healing | Spray | Active Ingredients - Benzethonium chloride 0.13%; Inactive Ingredients - Water, octoxynol-9, sodium citrate, polysorbate 20, propylene glycol, citric acid, diazolidinyl urea, polyquaternium-4, benzyl alcohol, methylparaben, fragrance |
| SensiCare | Skin Barriers/  Wound Healing | Spray | Silicone |
| Silverol | Topical antimicrobials | Cream | Silver sulfadiazine cream contains 1% w/w silver sulfadiazine. The vehicle in which the active ingredient is dispersed consists of water, stearyl alcohol, white petrolatum, polyoxyl 40 stearate, propylene glycol, isopropyl myristate, and sorbitan monooleate with 0.3% methylparaben as a preservative |
| Staquis | Anti-Eczema/  Anti-Itching | Ointment | EUCRISA contains crisaborole (active ingredient) and the following inactive ingredients: white petrolatum, propylene glycol, mono- and di-glycerides, paraffin, butylated hydroxytoluene, and edetate calcium disodiu |
| U Lactin | Moisturizers | Cream | Purified water, urea, mineral oil, petrolatum, triethanolamine, lactic acid, cetyl alcohol, propylene glycol stearate, sorbitan stearate, sodium lauryl sulfate, magnesium aluminum silicate, methylparaben, propylparaben |
| Xeracalm | Anti-Eczema/  Anti-Itching | Cream | Avène Thermal Spring Water, Glycerin, Mineral Oil, Cetearyl Alcohol, Oenothera Biennis Oil, Caprylic/Capric Triglyceride, Cetearyl Glucoside, Aquaphilus Dolomiae Extract, Arginine, Carbomer, Evening Primrose Oil/Palm Oil Aminopropanediol Esters, Glycine, Sodium Hydroxide, Tocopherol, Water |
| Zindaclin | Topical antimicrobials | Gel | Clindamycin phosphate 1%. Inactive ingredients: propylene glycol, ethanol, zinc acetate, hydroxyethylcellulose and sodium hydroxide |
